# 2-Methanesulfonamidobenzoic acid

**DOI:** 10.1107/S1600536809036113

**Published:** 2009-09-12

**Authors:** Muhammad Shafiq, Muhammad Zia-ur-Rehman, Islam Ullah Khan, Muhammad Nadeem Arshad, Imtiaz Ahmad

**Affiliations:** a Department of Chemistry, Government College University, Lahore 54000, Pakistan; b Applied Chemistry Research Centre, PCSIR Laboratories Complex, Ferozpure Road, Lahore 54600, Pakistan

## Abstract

In the title compound, C_8_H_9_NO_4_S, an intra­molecular N—H⋯O hydrogen bond gives rise to a six-membered ring. In the crystal structure, two mol­ecules are connected by O—H⋯O hydrogen bonds, forming a centrosymmetric dimer. These dimers are further connected by C—H⋯O hydrogen bonds.

## Related literature

For the synthesis and biological evaluation of sulfur-containing heterocyclic compounds, see: Zia-ur-Rehman *et al.* (2005 ▶, 2006 ▶, 2009 ▶); Xiao & Timberlake (2000 ▶); Lee & Lee (2002 ▶). For biological evaluation of sulfonamides, see: Hanson *et al.* (1999 ▶); Moree *et al.* (1991 ▶); Rough *et al.* (1998 ▶). For related literature on sulfonamides, see: Esteve & Bidal (2002 ▶); Soledade *et al.* (2006 ▶). For related structures, see: Gowda *et al.* (2007 ▶); Arshad *et al.* (2008 ▶).
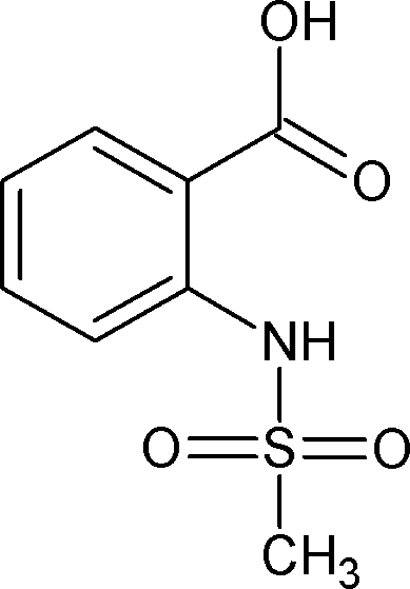


## Experimental

### 

#### Crystal data

C_8_H_9_NO_4_S
*M* *_r_* = 215.23
Triclinic, 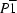

*a* = 5.2001 (2) Å
*b* = 8.6120 (4) Å
*c* = 11.2314 (5) Å
α = 72.675 (3)°
β = 84.155 (3)°
γ = 86.846 (3)°
*V* = 477.50 (4) Å^3^
*Z* = 2
Mo *K*α radiation
μ = 0.33 mm^−1^
*T* = 296 K
0.19 × 0.09 × 0.02 mm

#### Data collection

Bruker APEXII CCD area-detector diffractometer
Absorption correction: multi-scan (*SADABS*; Sheldrick, 1997 ▶) *T*_min_ = 0.941, *T*_max_ = 0.993
9379 measured reflections
2330 independent reflections
1261 reflections with *I* > 2σ(*I*)
*R*_int_ = 0.030

#### Refinement

*R*[*F* ^2^ > 2σ(*F* ^2^)] = 0.043
*wR*(*F* ^2^) = 0.121
*S* = 1.01
2330 reflections
131 parameters
H atoms treated by a mixture of independent and constrained refinement
Δρ_max_ = 0.21 e Å^−3^
Δρ_min_ = −0.19 e Å^−3^

### 

Data collection: *APEX2* (Bruker, 2007 ▶); cell refinement: *SAINT* (Bruker, 2007 ▶); data reduction: *SAINT*; program(s) used to solve structure: *SHELXS97* (Sheldrick, 2008 ▶); program(s) used to refine structure: *SHELXL97* (Sheldrick, 2008 ▶); molecular graphics: *PLATON* (Spek, 2009 ▶) and *Mercury* (Macrae *et al.*, 2006 ▶); software used to prepare material for publication: *SHELXTL* (Sheldrick, 2008 ▶) and local programs.

## Supplementary Material

Crystal structure: contains datablocks I, global. DOI: 10.1107/S1600536809036113/bt5054sup1.cif

Structure factors: contains datablocks I. DOI: 10.1107/S1600536809036113/bt5054Isup2.hkl

Additional supplementary materials:  crystallographic information; 3D view; checkCIF report

## Figures and Tables

**Table 1:** Hydrogen-bond geometry (Å, °)

*D*—H⋯*A*	*D*—H	H⋯*A*	*D*⋯*A*	*D*—H⋯*A*
O2—H2⋯O1^i^	0.78 (3)	1.89 (3)	2.671 (2)	172 (4)
N1—H1⋯O1	0.86	2.03	2.652 (2)	128
C4—H4⋯O3^ii^	0.93	2.37	3.235 (3)	155

Symmetry codes: (i) 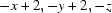; (ii) 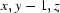.

**Fig. 1.**
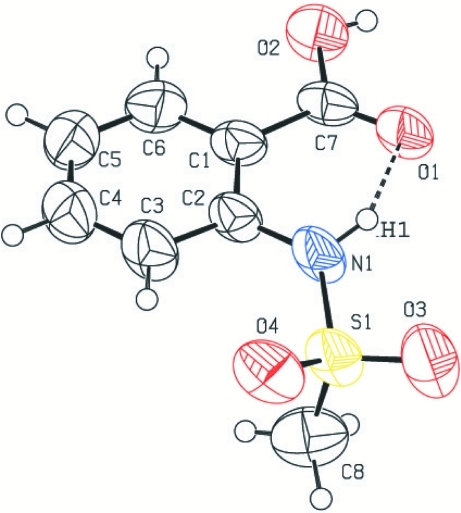
The molecular structure of the title compound, with displacement ellipsoids at the 50% probability level.

## supplementary crystallographic information

### Comment

Sulfonamides are well known for their enormous potential as biologically active molecules (Hanson *et al.*, 1999; Moree *et al.*, 1991; Rough *et al.*, 1998). Few of these are familiar as anti-hypertensive, anti-convulsant, herbicidal, anti-microbial and anti-microbial activities (Esteve & Bidal, 2002; Soledade *et al.*, 2006; Xiao & Timberlake, 2000; Lee & Lee, 2002). In the present paper, the structure of the 2-[(methylsulfonyl)amino]benzoic acid has been determined as a part of a research program involving the synthesis and biological evaluation of sulfur containing heterocyclic compounds (Zia-ur-Rehman *et al.*, 2005, 2006, 2009). In the molecule of (Fig. 1), bond lengths and bond angles are are almost similar to those in related molecules (Gowda *et al.*, 2007; Arshad *et al.*, 2008) and are within normal ranges. Each molecule exhibits an intramolecular N—H···O hydrogen bond which stabilizes the planar conformation and is linked to an adjacent one through head-to-tail pairs of O—H···O intermolecular interactions giving rise to dimeric motifs typical for carboxylic acids. Neighbouring dimers are further linked to each other through C—H···O interactions (Fig. 2).

### Experimental

A mixture of methyl 2-[(methylsulfonyl)amino]benzoate (1.0 g; 4.4 mmoles), sodium hydroxide (0.2 g; 5.0 mmoles) and water (30.0 ml) was stirred at room temperature for a period of one hour followed by addition of dilute hydrochloric acid to Congo Red (pH~5). Precipitates formed were filtered, washed with cold water and dried under vacuum followed by recrystallization in ethanol.

### Refinement

H atoms bond to C and N were placed in geometric positions (N—H = 0.86, C_aromatic_—H = 0.93, C_methyl_—H = 0.96 Å) using a riding model with *U*_iso_(H) = 1.2*U*_eq_(C,N,O) or *U*_iso_(H) = 1.5*U*_eq_(C_methyl_). The coordinates of the H atom bonded to O were refined.

### Crystal data

**Table:**

C_8_H_9_NO_4_S	*Z* = 2
*M**_r_* = 215.23	*F*(000) = 224
Triclinic, *P*1	*D*_x_ = 1.497 Mg m^−^^3^
Hall symbol: -P 1	Melting point: 373 K
*a* = 5.2001 (2) Å	Mo *K*α radiation, λ = 0.71073 Å
*b* = 8.6120 (4) Å	Cell parameters from 2313 reflections
*c* = 11.2314 (5) Å	θ = 2.6–26.6°
α = 72.675 (3)°	µ = 0.33 mm^−^^1^
β = 84.155 (3)°	*T* = 296 K
γ = 86.846 (3)°	Needle, colourless
*V* = 477.50 (4) Å^3^	0.19 × 0.09 × 0.02 mm

### Data collection

**Table:**

Bruker APEXII CCD area-detector diffractometer	2330 independent reflections
Radiation source: fine-focus sealed tube	1261 reflections with *I* > 2σ(*I*)
graphite	*R*_int_ = 0.030
φ and ω scans	θ_max_ = 28.3°, θ_min_ = 2.5°
Absorption correction: multi-scan (*SADABS*; Sheldrick, 1997)	*h* = −6→6
*T*_min_ = 0.941, *T*_max_ = 0.993	*k* = −11→11
9379 measured reflections	*l* = −14→14

### Refinement

**Table:**

Refinement on *F*^2^	Primary atom site location: structure-invariant direct methods
Least-squares matrix: full	Secondary atom site location: difference Fourier map
*R*[*F*^2^ > 2σ(*F*^2^)] = 0.043	Hydrogen site location: inferred from neighbouring sites
*wR*(*F*^2^) = 0.121	H atoms treated by a mixture of independent and constrained refinement
*S* = 1.01	*w* = 1/[σ^2^(*F*_o_^2^) + (0.0569*P*)^2^ + 0.0282*P*] where *P* = (*F*_o_^2^ + 2*F*_c_^2^)/3
2330 reflections	(Δ/σ)_max_ < 0.001
131 parameters	Δρ_max_ = 0.21 e Å^−^^3^
0 restraints	Δρ_min_ = −0.19 e Å^−^^3^

### Special details

**Table:**

Geometry. All e.s.d.'s (except the e.s.d. in the dihedral angle between two l.s. planes) are estimated using the full covariance matrix. The cell e.s.d.'s are taken into account individually in the estimation of e.s.d.'s in distances, angles and torsion angles; correlations between e.s.d.'s in cell parameters are only used when they are defined by crystal symmetry. An approximate (isotropic) treatment of cell e.s.d.'s is used for estimating e.s.d.'s involving l.s. planes.
Refinement. Refinement of *F*^2^ against ALL reflections. The weighted *R*-factor *wR* and goodness of fit *S* are based on *F*^2^, conventional *R*-factors *R* are based on *F*, with *F* set to zero for negative *F*^2^. The threshold expression of *F*^2^ > σ(*F*^2^) is used only for calculating *R*-factors(gt) *etc*. and is not relevant to the choice of reflections for refinement. *R*-factors based on *F*^2^ are statistically about twice as large as those based on *F*, and *R*- factors based on ALL data will be even larger.

### Fractional atomic coordinates and isotropic or equivalent isotropic displacement parameters (Å^2^)

**Table:**

	*x*	*y*	*z*	*U*_iso_*/*U*_eq_	
S1	0.13958 (10)	0.86241 (8)	0.36548 (6)	0.0718 (3)	
O1	0.7317 (3)	0.9547 (2)	0.09332 (14)	0.0702 (4)	
O2	0.9632 (3)	0.7901 (2)	0.00272 (17)	0.0884 (6)	
H2	1.047 (6)	0.868 (4)	−0.020 (3)	0.106*	
O3	0.1286 (4)	1.0325 (2)	0.3430 (2)	0.1179 (8)	
O4	−0.0938 (3)	0.7780 (2)	0.38190 (16)	0.0881 (6)	
N1	0.3205 (3)	0.8337 (2)	0.24607 (18)	0.0737 (6)	
H1	0.3768	0.9197	0.1899	0.088*	
C1	0.6130 (4)	0.6764 (3)	0.14278 (19)	0.0580 (6)	
C2	0.3943 (4)	0.6848 (3)	0.22472 (19)	0.0579 (6)	
C3	0.2556 (4)	0.5456 (3)	0.2809 (2)	0.0770 (7)	
H3	0.1102	0.5498	0.3355	0.092*	
C4	0.3279 (5)	0.4020 (3)	0.2577 (3)	0.0826 (7)	
H4	0.2305	0.3101	0.2959	0.099*	
C5	0.5438 (5)	0.3919 (3)	0.1784 (3)	0.0839 (8)	
H5	0.5945	0.2937	0.1635	0.101*	
C6	0.6818 (5)	0.5284 (3)	0.1222 (2)	0.0778 (7)	
H6	0.8272	0.5219	0.0682	0.093*	
C7	0.7712 (4)	0.8198 (3)	0.07879 (19)	0.0617 (6)	
C8	0.3072 (5)	0.7722 (4)	0.4963 (3)	0.0931 (9)	
H8A	0.4750	0.8186	0.4842	0.140*	
H8B	0.3260	0.6573	0.5079	0.140*	
H8C	0.2130	0.7910	0.5690	0.140*	

### Atomic displacement parameters (Å^2^)

**Table:**

	*U*^11^	*U*^22^	*U*^33^	*U*^12^	*U*^13^	*U*^23^
S1	0.0519 (3)	0.0691 (5)	0.0812 (5)	−0.0032 (3)	0.0323 (3)	−0.0144 (3)
O1	0.0596 (9)	0.0739 (11)	0.0679 (10)	−0.0159 (8)	0.0290 (7)	−0.0160 (8)
O2	0.0795 (11)	0.0919 (14)	0.0887 (13)	−0.0233 (9)	0.0491 (9)	−0.0336 (11)
O3	0.1275 (16)	0.0632 (12)	0.1399 (18)	−0.0009 (10)	0.0728 (14)	−0.0229 (11)
O4	0.0469 (8)	0.1114 (14)	0.0933 (13)	−0.0105 (8)	0.0251 (8)	−0.0194 (10)
N1	0.0676 (11)	0.0592 (12)	0.0772 (13)	−0.0082 (9)	0.0391 (10)	−0.0078 (10)
C1	0.0512 (11)	0.0722 (15)	0.0480 (12)	−0.0099 (10)	0.0099 (9)	−0.0167 (11)
C2	0.0486 (11)	0.0615 (14)	0.0558 (13)	−0.0077 (10)	0.0123 (9)	−0.0099 (10)
C3	0.0625 (13)	0.0719 (17)	0.0865 (18)	−0.0136 (12)	0.0254 (12)	−0.0161 (13)
C4	0.0825 (16)	0.0716 (17)	0.0894 (19)	−0.0235 (13)	0.0146 (14)	−0.0207 (14)
C5	0.0952 (19)	0.0732 (17)	0.0866 (18)	−0.0142 (14)	0.0148 (15)	−0.0340 (14)
C6	0.0761 (15)	0.0870 (19)	0.0708 (16)	−0.0099 (14)	0.0227 (13)	−0.0322 (14)
C7	0.0510 (11)	0.0813 (17)	0.0466 (12)	−0.0093 (11)	0.0156 (9)	−0.0146 (12)
C8	0.0601 (14)	0.132 (2)	0.089 (2)	−0.0001 (15)	0.0122 (13)	−0.0406 (18)

### Geometric parameters (Å, °)

**Table:**

S1—O3	1.4091 (18)	C2—C3	1.381 (3)
S1—O4	1.4164 (16)	C3—C4	1.363 (3)
S1—N1	1.6307 (18)	C3—H3	0.9300
S1—C8	1.740 (3)	C4—C5	1.376 (3)
O1—C7	1.223 (3)	C4—H4	0.9300
O2—C7	1.312 (2)	C5—C6	1.361 (3)
O2—H2	0.78 (3)	C5—H5	0.9300
N1—C2	1.400 (3)	C6—H6	0.9300
N1—H1	0.8600	C8—H8A	0.9600
C1—C6	1.384 (3)	C8—H8B	0.9600
C1—C2	1.401 (3)	C8—H8C	0.9600
C1—C7	1.477 (3)		
			
O3—S1—O4	119.14 (12)	C3—C4—C5	120.6 (2)
O3—S1—N1	104.71 (10)	C3—C4—H4	119.7
O4—S1—N1	109.48 (11)	C5—C4—H4	119.7
O3—S1—C8	109.48 (15)	C6—C5—C4	118.8 (2)
O4—S1—C8	107.25 (12)	C6—C5—H5	120.6
N1—S1—C8	106.10 (11)	C4—C5—H5	120.6
C7—O2—H2	107 (2)	C5—C6—C1	122.2 (2)
C2—N1—S1	127.27 (14)	C5—C6—H6	118.9
C2—N1—H1	116.4	C1—C6—H6	118.9
S1—N1—H1	116.4	O1—C7—O2	121.91 (19)
C6—C1—C2	118.5 (2)	O1—C7—C1	124.62 (18)
C6—C1—C7	119.46 (19)	O2—C7—C1	113.5 (2)
C2—C1—C7	122.1 (2)	S1—C8—H8A	109.5
C3—C2—N1	121.92 (19)	S1—C8—H8B	109.5
C3—C2—C1	118.7 (2)	H8A—C8—H8B	109.5
N1—C2—C1	119.34 (18)	S1—C8—H8C	109.5
C4—C3—C2	121.2 (2)	H8A—C8—H8C	109.5
C4—C3—H3	119.4	H8B—C8—H8C	109.5
C2—C3—H3	119.4		
			
O3—S1—N1—C2	179.0 (2)	C1—C2—C3—C4	−0.1 (4)
O4—S1—N1—C2	−52.2 (2)	C2—C3—C4—C5	0.7 (4)
C8—S1—N1—C2	63.2 (2)	C3—C4—C5—C6	−0.9 (4)
S1—N1—C2—C3	22.1 (3)	C4—C5—C6—C1	0.4 (4)
S1—N1—C2—C1	−158.81 (18)	C2—C1—C6—C5	0.3 (4)
C6—C1—C2—C3	−0.4 (3)	C7—C1—C6—C5	180.0 (2)
C7—C1—C2—C3	179.9 (2)	C6—C1—C7—O1	−177.7 (2)
C6—C1—C2—N1	−179.6 (2)	C2—C1—C7—O1	2.0 (4)
C7—C1—C2—N1	0.7 (3)	C6—C1—C7—O2	2.3 (3)
N1—C2—C3—C4	179.1 (2)	C2—C1—C7—O2	−177.9 (2)

### Hydrogen-bond geometry (Å, °)

**Table:**

*D*—H···*A*	*D*—H	H···*A*	*D*···*A*	*D*—H···*A*
O2—H2···O1^i^	0.78 (3)	1.89 (3)	2.671 (2)	172 (4)
N1—H1···O1	0.86	2.03	2.652 (2)	128
C4—H4···O3^ii^	0.93	2.37	3.235 (3)	155

Symmetry codes: (i) −*x*+2, −*y*+2, −*z*; (ii) *x*, *y*−1, *z*.
